# Assessment of source of information for polio supplementary immunization activities in 2014 and 2015, Somali, Ethiopia

**DOI:** 10.11604/pamj.supp.2017.27.2.10728

**Published:** 2017-06-09

**Authors:** Selamawit Yilma Bedada, Kathleen Gallagher, Aron Kassahun Aregay, Bashir Mohammed, Mohammed Adem Maalin, Hassen Abdisemed Hassen, Yusuf Mohammed Ali, Fiona Braka, Pierre M’pele Kilebou

**Affiliations:** 1 Expanded Program on Immunization, World Health Organization, Ethiopia; 2 Jigjiga University, Jigjiga, Ethiopia; 3 World Health Organization, Nigeria; 4 World Health Organization, Benin

**Keywords:** Polio, communication, social mobilization, independent monitoring

## Abstract

**Introduction:**

Communication is key for the successful implementation of polio vaccination campaigns. The purpose of this study is to review and analyse the sources of information utilized by caregivers during polio supplementary immunization activities (SIAs) in Somali, Ethiopia in 2014 and 2015.

**Methods:**

Data on sources of information about the polio campaign were collected post campaign from caregivers by trained data collectors as part of house to house independent monitoring. The sources of information analysed in this paper include town criers (via megaphones), health workers, religious leaders, kebele leaders (Kebele is the lowest administrative structure in Ethiopia), radio, television, text message and others. The repetition of these sources of information was analysed across years and zones for trends. Polio vaccination campaign coverage was also reviewed by year and zones within the Somali region in parallel with the major sources of information used in the respective year and zones. 57,745 responses were used for this analysis but the responses were received from < or = 57,745 individuals since some of them may provide more than one response. Moreover, because sampling of households is conducted independently during each round of independent monitoring, the same household may have been included more than once in our analysis. The methodology used for independent monitoring does not allow for the calculation of response rates. Monitors go from house to house until information from 20 households is received.

**Results:**

From the total 57,745 responses reviewed, over 37% of respondents reported that town criers were their source for information about the 2014 and 2015 polio SIAs. Zonal trends in using town criers as a major source of information in both study years remained consistent except in two zones. 87.5% of zones that reported at least 90% coverage during both study years had utilized town criers as a major source of information while the rest (12.5%) used health workers.

**Conclusion:**

We found that town criers were consistently the major source of information about the polio campaigns for Somali region parents and caregivers during polio immunization days held in 2014 and 2015. Health workers and kebele leaders were also important sources of information about the polio campaign for parents.

## Introduction

Polio is a highly contagious and potentially fatal infectious disease that can lead to paralysis [1–3]. There is no cure, but there are safe and effective vaccines. The eradication of polio is a top global health priority [4]. The strategy to eradicate polio is therefore based on preventing infection by immunizing every child until transmission stops and the world is polio free [5]. The polio eradication effort demands a communication approach that addresses the specific context of the country that enables the communities to be fully informed and actively participate in vaccination activities. It is also essential that the communication strategies are grounded in research [6].

The last indigenous case of wild poliovirus (WPV) in Ethiopia was confirmed in 2001, however, thereafter, several importations were experienced from neighboring infected countries. Ethiopia had been polio free for four years until August 2013 when a case of WPV was imported into the Somali region of Ethiopia from Puntland, Somalia. An additional 9 outbreak-associated cases were identified with the last case having onset of paralysis in January 2014. All cases occurred within the Doolo Zone of the Somali region making this area vulnerable for further WPV transmission. Supplementary immunization activities (SIAs), mass vaccination campaigns that aim to administer additional doses of oral poliovirus vaccine (OPV), are a key strategy of the Global Polio Eradication Initiative (GPEI). SIAs and routine immunization strengthening activities were implemented in Somali Region and other high risk areas of the country to increase population immunity against polio [7, 8]. Somali Region has a challenging geographic terrain with rural hard to reach areas, pastoralist communities and also an influx of refugees from Somalia. As part of outbreak response, Ethiopia implemented four National Immunization Days (NIDs) and 12 Subnational Immunization Days (SNIDs) between June, 2013 and September, 2015.

The Advisory Committee on Poliomyelitis Eradication (ACPE) recommended the implementation of independent monitoring of SIAs in all infected countries in November 2009 to rectify the problem of delayed and poor quality of SIA data provided by many countries as well as improve the credibility of the program [5, 7–9]. Independent monitoring of SIAs provide an objective measure of SIA quality that can be used to guide improvements to reach more children by enabling corrective action both during SIAs and in planning for the next vaccination campaign. Immunization decision making is not a straightforward process for parents. Information influences parental decision making on whether they immunize their child or not [9, 10]. The most common primary reason for non-vaccination is lack of awareness and misconception. In this regard, communication helps to provide health information to raise awareness, create and sustain demand, and encourage acceptance of vaccination services [11]. Immunization messages can be communicated through media, health workers, town criers, drama, and songs by local musicians [12]. Utilizing a communication channel which reaches the largest proportion of the target population is crucial to achieve desired success [13, 14]. Hence it is essential to review where parents receive their information about polio SIAs for strengthening its influence towards parental decision making. The parent's source of information regarding the polio campaign, which is the focus of our analysis, is one of the key SIA indicators to be assessed by independent monitoring teams. We conducted a review of the sources of information for polio SIAs in Somali Region to determine which approaches could be strengthened to improve demand and acceptance for polio vaccination.

## Methods

The World Health Organization (WHO) in Ethiopia coordinated the implementation of independent monitoring for all SIAs. Independent monitors travelled from house to house as well as to locations out of the household during the last two days of the SIA as well as for 2 days after the SIA was completed.

**Figure 1:**
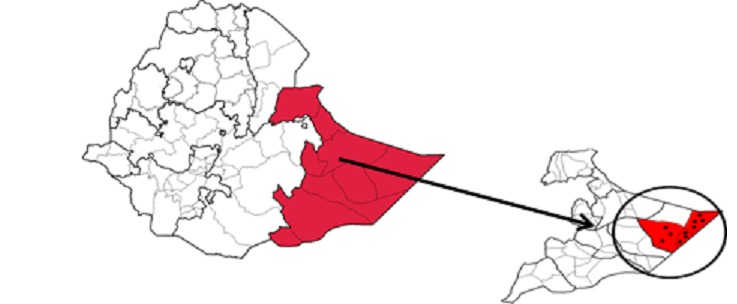
Location of WPV1 Cases, Dollo Zone, Somali Region, Ethiopia, 2013-2014

**Study Area:** the Somali Region found in Eastern Ethiopia has nine zones (i.e., Siti, Fafan, Jarar, Nogob, Shabele, Korahey, Afder, Liben, and Doolo) which cover an area of 350,000 square kilometres. Based on the 2007 census conducted by the Central Statistical Agency of Ethiopia (CSA) and inferring its growth rate, the Somali region had an estimated population 5 million in 2013, the majority of whom (90%) are pastoralists and agro-pastoralists [15] (Figure 1).

**Sampling:** sampling for the independent monitoring assessment was according to the global independent monitoring guideline which requires monitors to assess 20 households per kebele [7].

**Vaccination Coverage:** children who receive polio vaccine during the campaign have their left little fingers marked with indelible markers which last for several weeks. During independent monitoring, data collectors physically check to see if the children have been finger marked as a way to assess campaign performance [7]. The scope of this paper as mentioned in the topic was to study the 2014 and 2015 SIAs rounds hence the first 5 rounds conducted in 2013 were not included in this study. During 2014-2015, 11 SIAs and the corresponding 11 rounds of independent monitoring were conducted.

**Data Collection:** independent monitors collect information from parents or caretakers in households where children under 5 years old reside using a standardized data collection instrument. Information is collected on number of children in the household eligible to receive vaccine, number of children that are vaccinated and if not, the reason why they were not vaccinated. In addition, information is collected on sources of information used by parents and caregivers to convince them to vaccinate their children with oral poliovirus vaccine (OPV) during the SIAs.

**Data Analysis:** we conducted a descriptive analysis with the pooled quantitative data from polio SIAs conducted in 2014 and 2015 (SIAs rounds 6-16) in Somali Region. We analysed data on the major parental sources of information about the polio SIAs and trends in these data across the nine zones of Somali Region. The theme of our analyses related to where the respondents accessed information including single or multiple sources of information, how vaccine coverage was influenced by the sources of information in urban and rural settings, and the contribution of health workers, religious leaders, and kebele leaders as sources of information in 2014 and 2015 SIAs.

## Results

We analysed a total of 57,745 responses given in 2014 and 2015 by caretakers or parents of children targeted to receive OPV during polio SIAs in the Somali Region of Ethiopia. Respondents reported that the major source of information was megaphones used by town criers (37.6%), and the least common source of information were “other” sources (0.8%) (Table 1). Other major sources of information about Polio SIAs include health workers (24.3%) and kebele leaders (21.3%). When analysing the major sources of information across nine zones; out of the eight sources of information analysed in the data, town criers, kebele leaders, and health workers were the only three to be used repeatedly or at least once as a major source of information across the nine zones in 2014 and 2015.

**Table 1:** Major sources of information used in 2014 and 2015 polio SIA in Somali Region, Ethiopia

Sources of information used	Number (%) of respondents mobilized by the source					
	2014	%	2015	%	Total	%
Radio	4334	11.4	889	4.5	5223	9
Television	569	1.5	830	4.2	1399	2.4
Health workers	8445	22.2	5597	28.4	14042	24.3
Religious leaders	1452	3.8	640	3.3	2092	3.6
Kebele leaders	7602	20	4693	23.9	12295	21.3
Town Crier (Megaphone)	14804	38.9	6894	35	21698	37.6
Text message	482	1.3	79	0.4	561	1
Other	377	1	58	0.3	435	0.8
Total	38,065		19680		57745	

**Figure 2:**
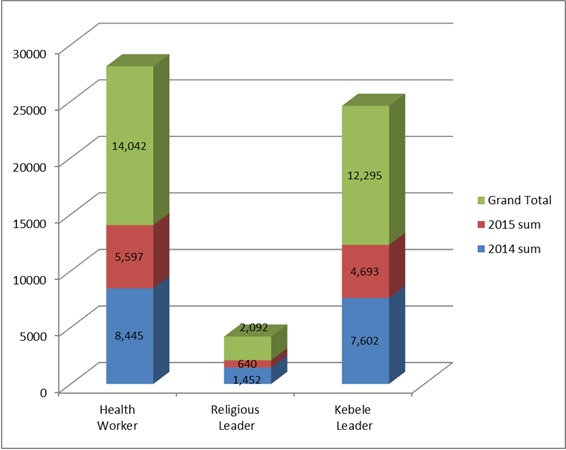
Contribution of health workers, religious leaders and kebele leaders as a source of information during Polio SIA, Somali Region, Ethiopia, 2014 and 2015

Table 2 shows OPV vaccination coverage in 2014 and 2015 in relation to the major sources of information used in different zones in the Somali region. Two zones had coverage of at least 90% and the other seven zones achieved less than 90% in 2014. In 2015, six zones had coverage of at least 90% whereas three zones remained below 90%. Figure 2 shows the contribution of health workers, kebele leaders, and religious leaders in 2014 and 2015 SIA in mobilizing people to immunize their children. Among the three groups health workers had the highest contribution (24.3%) to mobilizing parents and caretakers to vaccinate their children followed by kebele leaders (21.3%); religious leaders (3.6%) were reported to have the least contribution.

**Table 2:** Proportion of OPV vaccination coverage in 2014 and 2015 in relation to the major sources of information by zone, Somali Region, Ethiopia

Zone	Major source of information In 2014	% Coverage (Finger marked)	Major source of information In 2015	% Coverage (Finger marked)
**Afder**	Kebele leaders	88.6	Health workers	92.5
**Doolo**	Megaphone	86.6	Megaphone	95.6
**Fafan**	Megaphone	100	Megaphone	95.5
**Jarar**	Megaphone	88.4	Megaphone	87.3
**Korahe**	Megaphone	87.2	Megaphone	98.3
**Liben**	Megaphone	91.2	Megaphone	95.7
**Nogob**	Megaphone	86.7	Megaphone	96.2
**Shabelle**	Megaphone	89.8	Megaphone	86.9
**Sitti**	Megaphone	85.9	Health Workers	72.0

**Figure 3:**
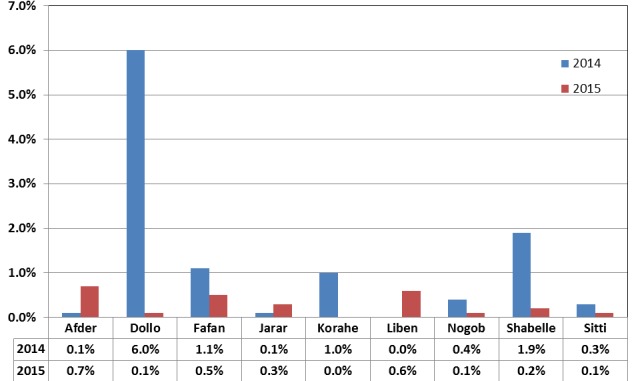
Zonal trend in using “other” sources of information about polio campaigns across the nine zones of the Somali region, Ethiopia, 2014-2015

Figure 3 shows the proportion of sources of information categorized as “other” across nine zones in 2014 and 2015. The proportion of “other” sources of information as compared to the rest of the seven sources was consistently reported to have low contribution (≤ 1.9%) in all zones for both years except in Doolo zone that reported 6% in 2014.

## Discussion

We found information provided by town criers through megaphones to be the major source of information for parents and caregivers in deciding to vaccinate their children with OPV; accounting for more than one third of the sources of information in both years that we reviewed. Town criers were consistently the major source of information in 2014 in eight zones and also in 2015 in six zones in Somali Region.

The OPV coverage during SIAs in the nine zones of the Somali Region improved in 2015 as compared to 2014 SIAs. The effect of information provided through megaphones as source of information for parents and caregivers could have contributed to the improved coverage in 2015, among other efforts. Town criers are selected from community level and are provided with a simple, translated, standard message that they broadcast using a megaphone as they move within the community starting seven days prior to and during the SIA implementation. Population distribution differs from place to place hence averagely 1,000 people would be targeted per town criers. The message alerts caregivers and parents of the polio campaign, dates and benefit of vaccination. Similar to our study, Waisbord and Larson in 2015 observed that the use of megaphones accounted for increased vaccination coverage in peri-urban and rural areas in Mozambique [16].

In our study, media was not at significant source of information for parents and caregivers in their decision to vaccinate their children, which is different from what Porter et al. reported in 2000 that higher exposure to media messages correlated with higher vaccination coverage rates in Russia in Novgorod city, Voronezh [17]. This may be explained by the population's low access to media in the Somali region along with the limited use of media in this region.

In our review, health workers had a higher contribution as source of information for parents' decision to vaccinate their children as compared to kebele leaders and religious leaders. This performance was similar across the numerous zones in both study years. In Somali region, health workers educate the family on immunization while they conduct house to house vaccination. In their study in 2014, Shen Angela, Fields Rebecca and et al from USA Johns Hopkins University- Global Health; Bloomberg School of Public Health, Center for Communication Programs found that, caregivers consistently cite health workers as their most important source of information about vaccination campaigns [18]. We also compared the above three sources with the contribution of the mass media used in 2014 and 2015 SIA. The contribution of health workers and kebele leaders is relatively better than mass media in 2014 and 2015 SIAs. However, the contribution of religious leaders from this analysis was not significant and was lower than the contribution of radio. This was in contrast to findings from a study in Pakistan which indicated that most parents believe that religious leaders could play an important role in motivating people to vaccinate their children [19]. Religious leaders' commitment may be challenged by the difficulty of accessing the population due to their scattered distribution and mobility. A study by UNICEF in the same intervention period in 2014 concluded that the dissemination of important messages and information is usually initiated and brokered by clan leaders and other community members of influence, including key cattle market traders and these were engaged to communicate polio related messages in Somali, Doolo Zone [20].

A study by Hill in London revealed that health workers who regularly visited individuals homes to promote health are credible sources of information that parents seek for their decision-making in immunization of their children (10). Political leaders and mass media have been shown to be trusted sources to address issues that impact the wellbeing of their community (11, 13). Our study had similar results for health workers and kebele leaders, which are the second and third contributors respectively, next to town criers, to impact parents to immunize their children but the religious leaders' and mass media results contrasts from the related studies mentioned above. We found that telephone text messaging was an infrequent source of information about the Polio SIAs. This could be due to low cell phone coverage in the region as well as the regularity of the implementation of this method of social mobilization.

Frieden reported that a community innovates based on actual existing practice to mobilize their people and implement public health programs and highlighted that such innovative practices need not be invented for each place but can be also adopted from other areas and be scaled up [21]. The finding from Doolo zone that reported a fairly high number of parents obtaining information from “other” sources may suggest a potential practice that could have been adapted to that area-specific context. A further qualitative study may be required to identify what other sources of information and social mobilization activities were being effectively used in Doolo zone.

Our analysis is subject to at least three limitations. First, we could not assess how appropriate the sources of information were to psychological or social factors or the different lifestyles of that community such as pastoralists, urban, and rural. Second, though the study sample was representative of all, the data reflects only where the IM team were able to reach and might not include very remote areas. Finally, we could not analyse the cost benefit of using these various sources of information because this information is not collected during independent monitoring. This limits our ability to recommend which sources of information would be most effective in urban, pastoralist and rural areas as well as their cost-effectiveness.

## Conclusion

In summary, we learned that information provided by town criers through megaphones was cited by the majority of parents and caregivers as one of the means through which they learned about the need for OPV. Health workers and kebele leaders were also reported as major sources of information about polio campaigns but the contribution of religious leaders was found to be not significant. We recommend that zones that obtained at least 90% OPV coverage and also used information through megaphones, as well as those who reported kebele leaders and health workers as a major source of information should share their experience to other low performing zones. This might be helpful not only for polio SIAs but also for implementing other routine immunization activities. Further qualitative studies should be undertaken to determine the specific characteristics of “other” sources of information in Doolo zone in 2014 to help understand if this zone used any different locally adaptable source of information for parents and caregivers in their decision to vaccinate their children.

### What is known about this topic

- The importance of communication before and during polio SIA to create demand for vaccination has been underlined on similar studies.

### What this study adds

- We reviewed and analysed the source of information used during Polio SIA in Ethiopia, Somali region using independent monitoring data collected during 11SIAs to learn where parents receive their information about polio SIAs for strengthening its influence towards parental decision making;
- We conducted a review of the sources of information for polio SIAs in Somali Region to determine which approaches could be strengthened to improve demand and acceptance for polio vaccination;
- Accordingly, we found that the effect of information provided through megaphones as source of information for parents and caregivers contributed to the improved coverage of polio SIA in Somali in this study period among other efforts.

## Competing interests

The authors declare no competing interests. The views expressed in the perspective articles are those of the authors alone and do not necessarily represent the views, decisions or policies of the institutions with which they are affiliated and the position of World Health Organization.
